# 2,4,6-Tribromophenol Interferes with the Thyroid Hormone System by Regulating Thyroid Hormones and the Responsible Genes in Mice

**DOI:** 10.3390/ijerph13070697

**Published:** 2016-07-12

**Authors:** Dongoh Lee, Changhwan Ahn, Eui-Ju Hong, Beum-Soo An, Sang-Hwan Hyun, Kyung-Chul Choi, Eui-Bae Jeung

**Affiliations:** 1 Laboratory of Veterinary Biochemistry and Molecular Biology, College of Veterinary Medicine, Chungbuk National University, Cheongju, Chungbuk 362-763, Korea; eastdaylight@gmail.com (D.L.); prac@naver.com (C.A.); 2 Laboratory of Veterinary Biochemistry, College of Veterinary Medicine, Chungnam National University, Daejeon 305-764, Korea; euijuhong@hotmail.com; 3 Laboratory of Biochemistry, Department of Biomaterial Science, College of Natural Resources and Life Science, Pusan National University, Miryang, Kyeongnam 50463, Korea; anbs@pusan.ac.kr; 4 Laboratory of Veterinary Biotechnology and Embryology, College of Veterinary Medicine, Chungbuk National University, Cheongju, Chungbuk 362-763, Korea; shhyun@chungbuk.ac.kr; 5 Laboratory of Biochemistry and Immunology, College of Veterinary Medicine, Chungbuk National University, Cheongju, Chungbuk 362-763, Korea; kchoi@chungbuk.ac.kr

**Keywords:** endocrine disruptor, 2,4,6-tribromophenol, thyroid hormone

## Abstract

2,4,6-Tribromophenol (TBP) is a brominated flame retardant (BFR). Based on its affinity for transthyretin, TBP could compete with endogenous thyroid hormone. In this study, the effects of TBP on the thyroid hormone system were assessed in mice. Briefly, animals were exposed to 40 and 250 mg/kg TBP. Thyroid hormones were also administered with or without TBP. When mice were treated with TBP, deiodinase 1 (*Dio1*) and thyroid hormone receptor β isoform 2 (*Thrβ2*) decreased in the pituitary gland. The levels of deiodinase 2 (*Dio2*) and growth hormone (*Gh*) mRNA increased in response to 250 mg/kg of TBP, and the relative mRNA level of thyroid stimulating hormone β (*Tshβ*) increased in the pituitary gland. *Dio1* and *Thrβ1* expression in the liver were not altered, while *Dio1* decreased in response to co-treatment with thyroid hormones. The thyroid gland activity decreased in response to TBP, as did the levels of free triiodothyronine and free thyroxine in serum. Taken together, these findings indicate that TBP can disrupt thyroid hormone homeostasis and the presence of TBP influenced thyroid actions as regulators of gene expression. These data suggest that TBP interferes with thyroid hormone systems

## 1. Introduction

The thyroid hormones, triiodothyronine (T3) and thyroxine (T4), are tyrosin-based hormones produced by the thyroid gland that are primarily responsible for the regulation of metabolism [1]. The secretion of hormones is regulated by thyroid-stimulating hormone (TSH). Like other hormones, thyroid hormones bind to several blood proteins such as albumin, thyroxine-binding globulin, and transthyretin [2]. While thyroid hormone levels and binding affinity to blood protein differ among species, the unbound form of T3 in the blood is believed to be the active form [3]. T3 then binds to thyroid hormone receptor (THR) in target cells to regulate thyroid dependent genes [4]. Expression of THR is tissue dependent, and its activity differs by subtype. There are two major isoforms in mouse, THR α, which is distributed in many various tissues, and THR β, which is expressed in the brain and pituitary gland [5]. Endocrine system disruption, especially associated with estrogenic compounds, is well known and reported. In addition, thyroid, adrenal, neuroendocrine, and reproductive systems have also recently been identified as targets for endocrine disruptors. Bisphenol A, a well-known weak estrogen agonist [6,7], was recently shown to influence thyroid-related systems, as well as to hinder thyroperoxidase activity and act as a Thr antagonist [8]. Brominated flame retardants (BFRs) are well known for their ability to interrupt thyroid function because of their structural similarity to thyroid hormones. BFRs have also been reported to interfere with thyroid function by regulating various thyroid hormone mediated proteins, such as sodium iodide symporter, thyroperoxidase, thyroxine-binding globulin, transthyretin, deiodinase and THR. Polybrominated diphenyl ether (PBDE), one of the BFRs, has been continuously researched for its association with THs [9,10,11].

After target cells uptake thyroid hormones, they deiodinate thyroid hormones, and this process is an important thyroid system pathway. Deiodinase II (*Dio*2) removes one iodide from the outer ring, mainly activating T3 from T4. Deiodination of the inner ring is performed by Deiodinase III (*Dio*3), thus making the inactive form of reverse triiodothyronine. Removal of iodine from both the outer and inner ring of thyroid hormones is mediated by deiodinase I (*Dio1*) [9,10]. *Dio1* or *Dio2* are thyroid hormone response genes which expression is dependent on tissue-specific expression of receptor subtypes. Hypothalamic-pituitary-thyroid gland (HPT) axis is regulated carefully. When the body needs thyroid hormones, thyrotropin-releasing hormone (TRH) is secreted from the hypothalamus. TRH induces thyroid stimulating hormone (TSH) secretion by affecting thyrotrophic cells in the adenohypophysis. TSH increases production of thyroid hormones in the thyroid gland. Its production is also dependent on iodide uptake in the thyroid gland. Total hormone or free hormone and thyroid stimulating hormone levels are indicators of thyroid status of the body [12]. The levels of these hormones modulate the expression of marker genes. Tissue-specific expression of THR and *Dio* is regulated by serum thyroid hormone levels. Enzyme activity of *Dio1* is highly induced by T3 in rodent liver, while THR is suppressed by thyroid hormones. These negative or positive feedback systems are essential for regulating the blood or tissue thyroid hormone levels. Induction of *Dio1* is thought to participate in transforming T4 into rT3 or T3 to 3,3’-diiodothyronine (D2), thus suppressing hormone activity in the tissues. THR decrease in target tissues hinders the hormone entrance to cells [5,12]. 

BFRs are essential to plastics that are required to endure high temperatures. For example, they are widely used in electrical equipment, polyurethane foams and antifungal agents. Although some harmful BFRs have been banned in a few countries, similar substitutions are being continuously produced, and their potential effects are not currently known. 2,4,6-Tribromophenol (TBP) is a BFR that has been identified as a thyroid hormone disruptor. In the United States, over 23,000 tons of TBP were produced in 2006, while 3600 tons were produced in Japan in 2001 [13,14]. A previous study showed that TBP had a high binding affinity to transthyretin and did not bind to THR in vitro [15]. Chronic exposure of zebrafish to TBP impaired reproduction. TBP was also shown to have adverse effects against estrogen and androgen levels [16]. Following exposure to TBP, several genes were up-regulated in females, while they were down-regulated in males because thyroid hormones have the opposite effect on estrogen response gene expression [17]. However, others have reported that TBP does not regulate estrogen response genes or proliferation [18,19]. Although the effects of TBP on estrogen and androgen have been examined, their effects on the thyroid system have not been thoroughly investigated. Therefore, this study was conducted to investigate the effects of TBP on the thyroid system.

## 2. Experimental Section

### 2.1. Chemicals

T3, T4 and TBP were purchased from Sigma-Aldrich (St. Louis, MO, USA). Stock solutions were made by dissolving chemicals in dimethyl sulfoxide (DMSO; Santa Cruz Biotechnology, Santa Cruz, CA, USA) and diluting them with corn oil (Sigma-Aldrich) when needed.

### 2.2. Animals

Post-natal day (PND) 14 female ICR mice with a dam were purchased from SAMTAKO (Gyeonggi-do, Korea) and allowed to acclimate for 7 days. Each group of five mice (*n* = 5) was breed in a polycarbonate cage with non-phytoestrogen beta chip bedding. To eliminate xenoestrogenic effects other than those exerted by the drugs administered, a non-steroid pellet diet AIN-76A (Central Lab Animal Inc., Seoul, Korea) and sterile water were provided ad libitum. The temperature of the environment was set at 20 °C–24 °C with 40%–60% relative humidity and a 12-h light-dark cycle. A 1000 mg/kg dose of TBP killed half of the mice in the groups. Therefore, two doses of TBP (40, 250 mg/kg) were administered at PND 21 days. TBP is generally used as a flame retardant, so exposure to TBP via the GI tract is much rarer than other forms of exposure. Therefore, to avoid first pass effect, we used subcutaneous injection rather than oral administration for 20 days. T3 (100 μg/kg) and T4 (100 μg/kg) were also administered or not TBP (250 mg/kg) to evaluate the interference effect of TBP with thyroid hormone. The dose of TBP were determined due to IUCLID dataset, and extrapolate by extrapolation equation [20]. The animals were sacrificed 1 day after the last administration. The Institutional Animal Care and Use Committee (IACUC) of Chungbuk National University approved all animal experimental procedures (Approval No. CBNUA-866-15-01).

### 2.3. Quantitative Real-Time qPCR

Mice were sacrificed by cervical dislocation. Organs were washed with cold sterile saline and homogenized in TRIzol (Life Technologies, Carlsbad, CA, USA) with a bullet blender (Next Advance, Averill Park, NY, USA). Total RNA was extracted from the homogenate according to the manufacturer’s instructions. The integrity of total RNA was confirmed by the 28S/18S and 5S rRNA integrity with electrophoresis. Total RNA concentration was measured, and 1 μg of RNA was reverse transcribed using Moloney murine leukemia virus (mMLV) reverse transcriptase (iNtRON Bio, Gyeonggi-do, Korea) with a random 9-mer primer (TaKaRa Bio Inc., Shiga, Japan) to produce first-strand complementary DNA (cDNA). 1 μL of cDNA template was added to 10 μL of 2xSYBR Premix Ex Taq (TaKaRa Bio Inc.) and 10 pmol of each specific primer. qPCR was performed under the following conditions: 40 cycles of denaturation at 95 °C for 30 s, annealing at 60 °C for 30 s, and extension at 72 °C for 30 s. The threshold cycle (CT) value was determined automatically during the exponential phase of the delta CT fluorescence detection graph. 18S RNA (18S) was used as an endogenous reference, while deiodinase 1 (*Dio1*), deiodinase 2 (*Dio2*), thyroid hormone receptor β isoform 2 (*Thrβ2*), thyroid hormone receptor β isoform 1 (*Thrβ1*), thyroid stimulating hormone β (*Tshβ*) and growth hormone (*Gh*) were the genes of interest (GOI). Relative quantification was based on the comparison of CT at a constant fluorescent intensity. The amount of transcript is inversely related to the observed CT, and for every twofold dilution in the transcript, CT is expected to increase by 1. Relative expression was calculated using the equation *R* = 2^−(ΔCTsample − ΔCTcontrol)^.

### 2.4. Histology

Thyroid gland was dissected along with the trachea and fixed with 4% neutral buffered formalin for 1 day. Tissue processing was performed using a Tissue-Tek VIP 5 system (Sakura, Torrance, CA, USA) according to the manufacturer’s instructions. Paraffin block and tissue slides were generated using a LEICA (Wetzlar, Germany) EG1150H and LEICA RM2255, respectively. Slides contained two thyroid glands, and each slide was sectioned at 4 μm. Hematoxylin & eosin staining was conducted according to the general protocols, after which samples were mounted with mounting medium (Sigma-Aldrich). To evaluate the activity of the thyroid gland, the surface area of the thyroid follicle and epithelium was measured using the ImageJ software with five slides for each group. The volume densities of thyroid follicles, follicular epithelium, colloid, interfollicular tissue and capillary network were measured, and calculated activation index of the thyroid gland. The activation index, which represents the ratio of the volume density of follicular epithelium to the volume density of colloid was introduced by Kalisnik [21]. All histological and stereological analyses were made by the same researcher.

### 2.5. Serum Hormone Analysis

Blood was collected from the abdominal vena cava and clotted at room temperature for 1 h. Clotted blood was centrifuged at 2000 g for 15 min at 4 °C. Next, the supernatant was carefully drained and stored at −80 °C until analysis. The serum levels of free T3 and T4 were measured using LKF31 and LFT42 kits, (Siemens, Berlin, Germany). This hormone assay is a direct or single test assay, and its results are interpolated from a stored standard curve calibrated in terms of free T3/T4 concentrations. All procedures for this assay were processed according to the manufacturer’s instructions. The value of hormones was analyzed by an Immulite 1000 small bench top immunoassay analyzer (Siemens).

### 2.6. Statistical Analysis

Bar graphs are presented as the means ± standard deviation (SD) and box and whisker graph are presented with the median, mean, 1.5 interquartile range and outliers. Experimental results were analyzed by one-way ANOVA and Tukey’s studentized range test. Statistical analyses were performed using SAS (version 9.2; SAS Institute, Cary, NC, USA). *p* values < 0.05 were considered significant.

## 3. Results

### 3.1. The mRNA Expression of Thyroid Hormone-Related Genes in the Pituitary Gland

To investigate the effects of TBP in the mouse pituitary gland, we observed several genes related to thyroid hormones. Mice were exposed to TBP with or without thyroid hormones to investigate the competitive effects of TBP with blood transthyretin. 

**Figure 1:**
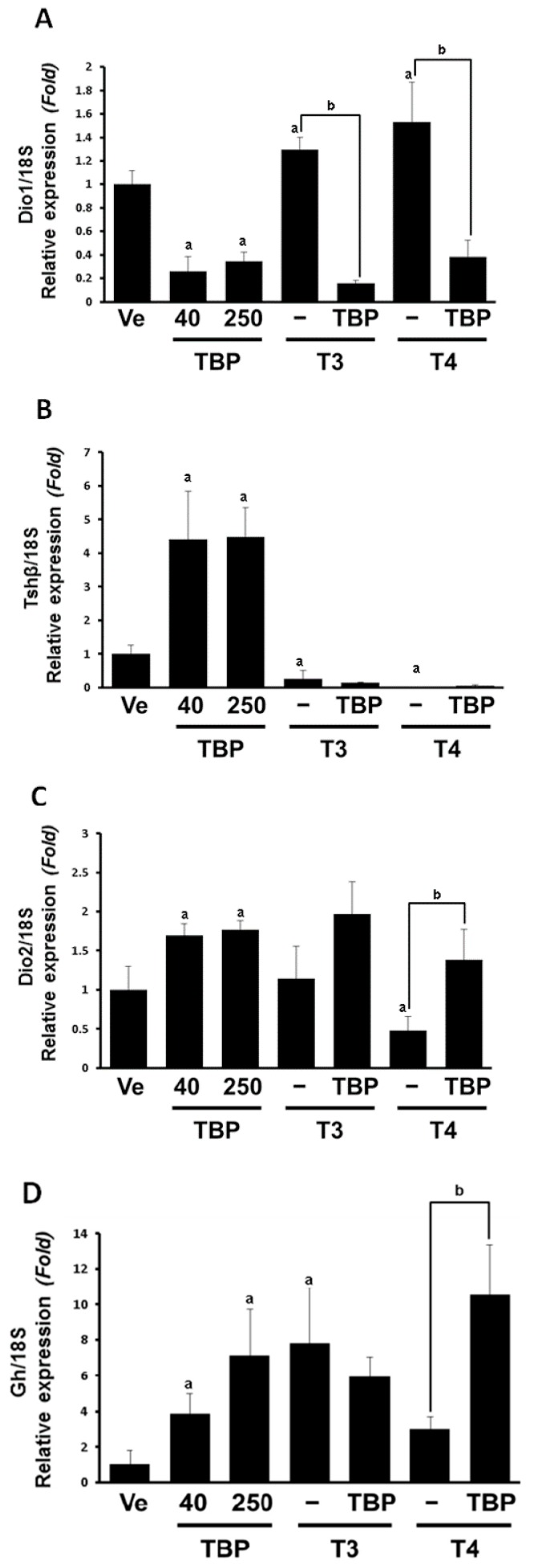

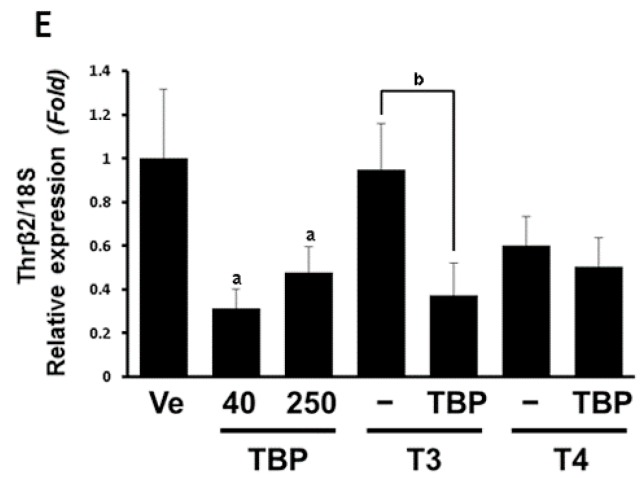
Expression levels of *Dio1* (**A**); *Dio2* (**B**); *Gh* (**C**); *Tshβ* (**D**) and *Thrβ2* (**E**) mRNA in the pituitary gland. Results presented in the bar graph are divided according to chemical administered and subdivided according to the presence or absence of 2,4,6-tribromophenol. Ve, vehicle; T3, triiodothyronine; T4, thyroxine; TBP, 2,4,6-tribromophenol. ^a^ *p* < 0.05 versus vehicle, ^b^ *p* < 0.05 versus without TBP. Data are presented as the mean ± SD.

The levels of a down-regulated indicator of thyroid hormones, *Dio1*, in the pituitary gland decreased by over 80% in response to both 40 and 250 mg/kg of TBP. In the presence of the extra thyroid hormones, T3 and T4, the repressive effects of TBP were further accentuated (Figure 1A). In contrast to *Dio1* expression, *Dio2* increased in the TBP treated groups (40 and 250 mg/kg). As expected, *Dio2* mRNA decreased significantly in response to treatment with T4 (0.47-fold compared to Ve group) alone, but this decrease was reversed when the samples were also treated with TBP. The mRNA levels of *Dio2* were not significantly changed in the T3-treated group with and without TBP (Figure 1B). In the growth stage, thyroid hormones stimulate the secretion of *Gh* in the pituitary gland. We observed robust (4-fold and 7-fold) increases of *Gh* mRNA levels in response to 40 mg/kg TBP and 250 mg/kg TBP, respectively, compared to the control group. The expression of *Gh* mRNA was also induced by T3 and T4, with a greater response being observed in T3 (Figure 1C). While T3-induced *Gh* mRNA was not affected by co-treatment with TBP, robust increases (10-fold) were observed in response to combined treatment with TBP and T4 (Figure 1C). As shown in Figure 1D, *Tshβ* mRNA expression was reduced in the T3 or T4 treated group, suggesting negative feedback on TSH. The co-treatment effects of TBP with T3 and T4 were not comparable (Figure 1D). In parallel with *Dio1*, *Thrβ2* mRNA was decreased by over 60% in response to both 40 and 250 mg/kg TBP. However, the effects of T3 and T4 on *Thrβ2* were not significant (Figure 1E).

### 3.2. The mRNA Expression of Dio1 and Thrβ1 in the Liver

**Figure 2:**
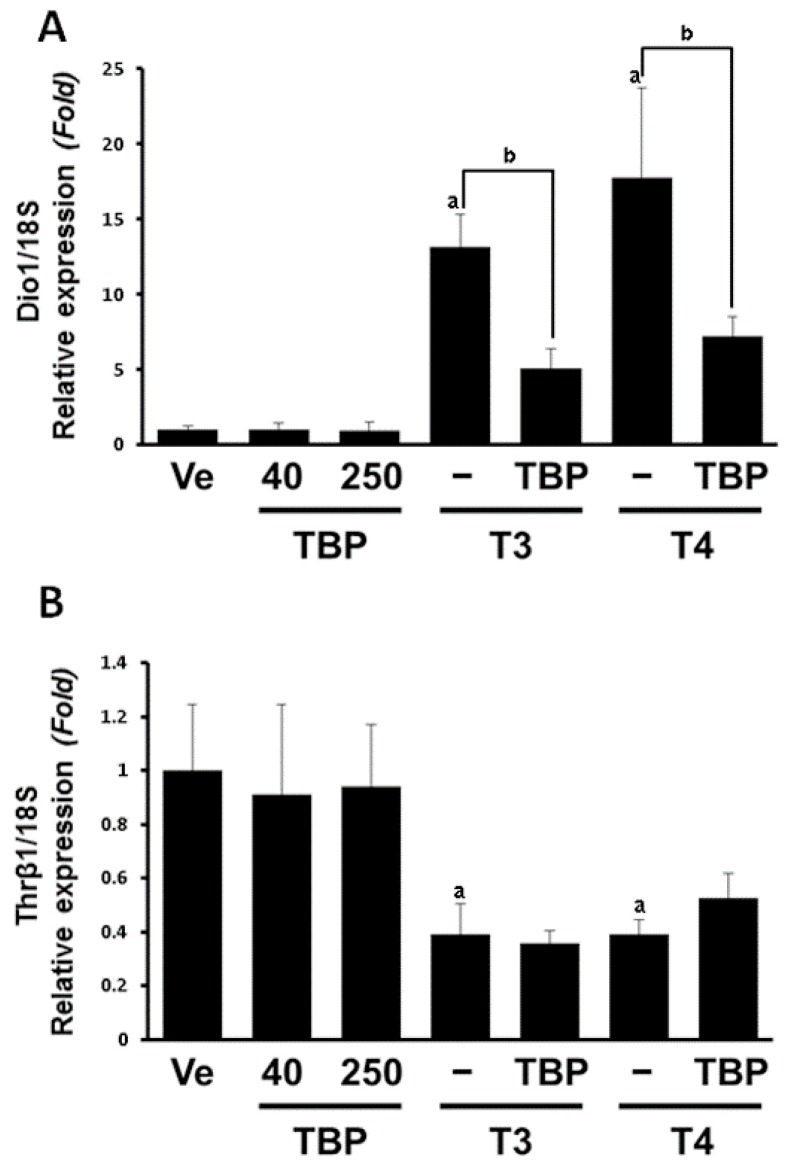
Expression levels of *Dio1* (**A**) and *Thrβ1* (**B**) mRNA in liver. Results presented in the bar graph are divided according to chemical administered and subdivided according to the presence or absence of 2,4,6-tribromophenol. Ve, vehicle; T3, triiodothyronine; T4, thyroxine; TBP, 2,4,6-tribromophenol. ^a^ *p* < 0.05 versus vehicle, ^b^ *p* < 0.05 versus without TBP. Data are presented as the mean ± SD.

To assess the effects of TBP in other tissues, we monitored thyroid hormone-related genes in the liver, which is responsible for most enzymatic regulation of hormones, as well as production and secretion of some thyroid hormone binding proteins. TBP alone did not significantly change the *Dio1* mRNA levels. However, both T3 (14-fold) and T4 (18-fold) highly upregulated *Dio1* expression, while this induction was significantly repressed when TBP was coadministered (Figure 2A). As a major subtype of thyroid hormone receptor in the liver, *Thrβ1* expression was tested. *Thrβ1* transcripts were not affected by TBP in the absence or presence of T3 and T4. However, *Thrβ1* mRNA was suppressed in the T3 (0.39-fold) and T4 (0.39-fold) treated group (Figure 2B).

### 3.3. Morphological and Histological Analysis of Thyroid Gland

**Figure 3:**
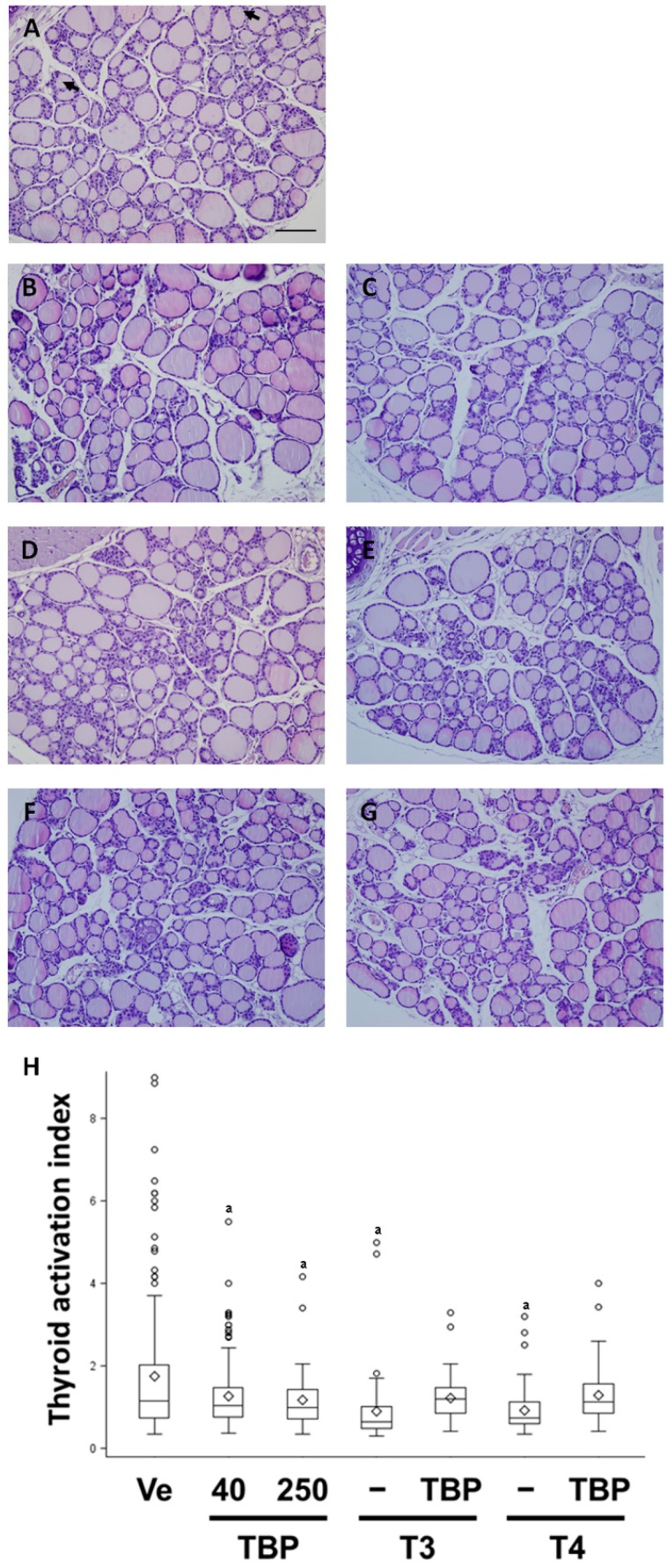
Thyroid gland histology ((**A**) Ve; (**B**) TBP 40; (**C**) TBP 250; (**D**) T3; (**E**) TBP-T3; (**F**) T4; (**G**) TBP-T4) and thyroid gland activation index (**H**). Arrow indicates resorption lacunae of follicle. Each thyroid gland was pictured at 200× magnification. Scale bar represents 100 μm. Results presented in the box and whisker graph are divided according to chemical administered and subdivided according to the presence or absence of 2,4,6-tribromophenol. Ve, vehicle; T3, triiodothyronine; T4, thyroxine; TBP, 2,4,6-tribromophenol. ^a^ *p* < 0.05 versus vehicle, ^b^ *p* < 0.05 versus without TBP. Data are presented as the 1.5 interquartile range, mean (diamond) and outlier (circle).

To assess the morphological and histological change by TBP, five slides/group were analyzed. Observation of the follicle shape, follicular epithelium, mesenchyme and adjunct blood vessels did not reveal great changes in the morphology of the thyroid gland (Figure 3). However, indicators of thyroid gland activity (size and number of resorption lacunae, carrying thyroglobulin) were decreased in T3 and T4 treated groups, although they varied in the TBP treated groups (Figure 3A–G). 

The morphology of the thyroid gland changed when the function was altered by various factors. These morphologies, including follicular volume or epithelial shape, are major indicators of thyroid gland function. Adjunct connective tissue and blood vessels are also important indicators of thyroid gland function. During production of thyroglobulin, follicles of active thyroid glands are much larger than those of normal or inactive glands. In this study, the thyroid activation index calculated from follicle size varied among groups. Thus, data were presented as box and whisker plots. Partially large follicles and reduced epithelia were observed in the thyroid hormone and TBP group and the co-treatment group. Thus, the overall thyroid activation index was reduced in the thyroid hormone (T3 for 0.78-fold, T4 for 0.40-fold) and TBP (40 mg/kg for 0.62-fold, 250 mg/kg for 0.78-fold)group, as well as the co-treatment group when compared with the vehicle control group (Figure 3H).

### 3.4. Concentration of Free T3 (fT3) and T4 (fT4) Levels in Serum

The levels of T3 and T4 were assessed in mouse sera after exposure to endogenous thyroid hormones or exogenous TBP compound. Due to the limited volume acquired from mouse serum, only free T3 and T4 were analyzed in this study. The fT3 and fT4 levels were decreased in a dose-dependent manner in response to TBP exposure, whereas fT3 was not detected in T3 treated mice (Table 1). Interestingly, when mice were treated with T3, fT4 was significantly increased, while TBP repressed the increase. T4 with or without TBP had a similar effect as T3 on the level of fT4. T4 alone did not significantly alter fT3 concentrations, although TBP reduced fT3 when combined with T3.

## 4. Discussion

Endocrine disturbances occur in various hormone systems, particularly the gonads. Estrogenic compounds are more common and well established as endocrine disruptors [22,23], and evaluation of these compounds has been well documented in the Endocrine Disruptor Screening Program (EDSP) and OECD. However, endocrine disruptors that can interfere with thyroid hormone have not been investigated as thoroughly as estrogenic compounds.

Based on recent study, brominated flame retardants (BFR) was examined for their thyroid function interrupting ability. BFRs was reported to interfere with thyroid function by regulating various thyroid hormone mediated proteins, such as sodium iodide symporter, thyroperoxidase, thyroxine-binding globulin, transthyretin, deiodinase and THR. In this study, TBP, which is regarded as a relatively harmful flame retardant, was assessed for its endocrine disrupting abilities.

Thyroid hormones affect nervous tissue development and maintenance of general tissue functions. For maintaining homeostasis of thyroid hormone system, deiodination is an essential step in thyroid hormone metabolism [12]. There are three subtypes of deiodinase, and their expression and activities are known to be tissue specific. In the pituitary gland, *Dio2* primarily activates T3 from T4 by removing one iodide from the outer ring of T4. *Dio1* removes both the outer and inner rings of iodine from thyroid hormones. *Dio*s are used as markers of thyroid hormone status or laboratory research. *Dio*s regulate thyroid activity depending on their isoform. TSH is a primary factor for stimulating the thyroid gland. GH is a secreted protein in the pituitary gland that responds to thyroid hormone to increase the metabolism in the body. THR is also regulated by thyroid hormone. In this study, administration of thyroid hormones led to up-regulation of *Dio1* transcript in the pituitary to inactivate the thyroid hormone. *Dio1* transcripts are known as thyroid hormone target genes in pituitary glands [24]. Administration of TBP was found to down-regulate *Dio1*, and reduction of *Dio1* expression inhibited turnover of T4 to T3. Increased *Dio1* expression in response to T3 and T4 in the pituitary gland results in activation of T4 to T3. However, the main subtype of the pituitary gland was *Dio2*, which showed the opposite expression pattern from *Dio1* [25], being decreased by T4 treatment and increased by TBP treatment. The down-regulation of *Dio1* and up-regulation of *Dio2* by TBP suggests that TBP had an anti-thyroid effect on the pituitary gland [26]. Co-treatment with TBP inhibited thyroid hormone T3 and/or T4 action and altered expression of the thyroid hormone-responsive *Dio1* and *Dio2* genes. *Dio1* is a major subtype in the liver that is also induced by T3 and T4 [27]. TBP treatment alone showed no significant effect on *Dio1* expression in the liver, while inhibition of T3 and T4 induced up-regulation of *Dio1*. The subsequent enzyme activity of *Dio1* was also highly induced by T3 in rodent liver in a previous study [28].

After identifying the thyroid hormone-disrupting abilities of TBP at the deiodinase transcript level, we examined other thyroid hormone responsive genes in the pituitary gland (*Tshβ*, *Thrβ2* and *Gh*). Increased thyroid hormones are bound to *Thrβ2* and decrease thyroid stimulating hormone (TSH) secretion from the pituitary gland by negative feedback in response to thyroid hormone [29,30]. Thus, the decreased level of *Thrβ2* in the present study suggests that TBP acted like an anti-thyroid hormone. While *Tshβ* expression decreased in response to thyroid hormones and increased after TBP treatment, TBP did not inhibit the effects of thyroid hormone when administered together. Interestingly, *Gh* was induced by TBP, suggesting that TBP could behave like a thyroid hormone.

To synthesize thyroid hormone from follicles, the volume of epithelial cells surrounding the colloid increases. Otherwise, the colloid volume decreases due to uptake of thyroglobulin by epithelial cells. Thus, the activity of the thyroid gland is assumed to be a constant of epithelial volume division colloid volume. These mechanisms are mediated by several proteins expressed in the thyroid gland. Sodium iodide symporter (NIS) plays a major role in storage of iodide in follicular cells. Sodium and iodide ions from blood are taken up by NIS using ATP. Iodination of thyroglobulin (TG) by thyroperoxidase (TPO) is also important to thyroid function [31]. Iodide extruded to colloids is combined with exocytosed TG by TPO [32]. These iodized TG are endocytosed to follicular cells and cleaved to form thyroid hormones. The thyroid gland activity index decreased in response to thyroid hormone and TBP, but was not further decreased by TBP when treated with thyroid hormones.

Thyroid function was analyzed by measuring serum free thyroid hormones because free hormones are more accurately reflected than total hormones [33]. While fT3 was decreased by T3 and TBP, fT4 was decreased by TBP with or without thyroid hormones. Similar to the T3 treated group, serum fT3 was reduced in a dose-dependent manner in response to TBP treatments. T4 treatment does not alter the fT3 levels. Opposite regulation of fT4 was shown in TBP when compared with thyroid hormones. Interestingly, fT3 and fT4 were down regulated after T3 and T4 administration. It is possible that long-term treatment of the hormones caused negative feedback regulation. It was previously reported that T3 and T4 treatment lowered serum TSH levels and induced unsteady levels of the hormone [34]. Other study, also confirmed that BFR mixture reduced the serum T4 level to 73% of control levels at the highest dose for their experiment (20 mg/kg dosage) [35]. Opposite effect of TBP on the thyroid system was also reported. For example, Norrgran et al., demonstrated that feline hyperthyroidism was induced by brominated phenolic compounds [36]. In summary, we evaluated the thyromimetic effect of TBP in the thyroid gland based on genetic and pathological changes, but the accumulation of TBP in tissues or blood requires further examination.

## 5. Conclusions

In conclusion, TBP regulated TSH followed by serum fT3 and fT4, which again regulated thyroid hormone target genes. These findings suggest that TBP is an endocrine disrupting chemical that acts by interfering with the thyroid hormone system in mice.

## Figures and Tables

**Table 1:** Free triiodothyronine and free thyroxine levels in serum.

Hormones	Ve	TBP (mg/kg)		T3 (100 μg/kg)		T4 (100 μg/kg)	
	-	40	250	-	TBP (250 mg/kg)	-	TBP (250 mg/kg)
Free T3 (pg/mL)	1.64 ± 0.16	1.33 ± 0.22 ^a^	0.93 ± 0.06 ^a^	<0.3 ^a^	<0.3	1.64 ± 0.24	0.73 ± 0.09 ^b^
Free T4 (ng/dL)	1.80 ± 0.24	1.21 ± 0.22 ^a^	1.30 ±0.23 ^a^	2.33 ± 0.27 ^a^	1.99 ± 0.11 ^b^	2.76 ± 0.24 ^a^	1.62 ± 0.17 ^b^

The results presented in the table are divided according to chemical administered and subdivided according to the presence or absence of 2,4,6-tribromophenol. Ve, vehicle; T3, triiodothyronine; T4, thyroxine; TBP, 2,4,6-tribromophenol. ^a^ *p* < 0.05 versus vehicle, ^b^ *p* < 0.05 versus without TBP. Data are presented as the mean ± SD.
